# End-point diagnostics of *Giardia duodenalis* assemblages A and B by combining RPA with CRISPR/Cas12a from human fecal samples

**DOI:** 10.1186/s13071-024-06559-0

**Published:** 2024-11-12

**Authors:** Yilin Wang, Fuchang Yu, Yin Fu, Qian Zhang, Jinfeng Zhao, Ziyang Qin, Ke Shi, Yayun Wu, Junqiang Li, Xiaoying Li, Longxian Zhang

**Affiliations:** 1 College of Veterinary Medicine, Henan Agricultural University, Zhengzhou, Henan 450000 People’s Republic of China; 2 National International Joint Research Center for Animal Immunology, Zhengzhou, 450000 Henan People’s Republic of China; 3 College of Animal Science and Technology, Tarim University, Alar, 843300 Xinjiang People’s Republic of China; 4 Yebio Bioengineering Co., Ltd of Qingdao, Qingdao, 266108 Shandong People’s Republic of China; 5 School of Medicine, Xinxiang University, Jinsui Road 191, Xinxiang, 453003 People’s Republic of China

**Keywords:** *Giardia duodenalis*, Recombinase polymerase amplification, CRISPR/Cas12a, Visualized detection, On-site detection

## Abstract

**Background:**

*Giardia duodenalis* is a common enteric protozoan parasite that is categorized into eight assemblages (A–H). In particular, assemblages A and B are zoonotic, capable of infecting both humans and animals worldwide, resulting in significant economic losses and public health challenges in epidemic regions. Thus, the development of rapid, accurate and non-laboratory-based diagnostic methods for infected animals is crucial for the effective prevention and control of giardiasis. Recent advancements in clustered, regularly interspaced, short palindromic repeats (CRISPR) and CRISPR-associated (Cas) protein (Cas12a) systems allow promising avenues for nucleic acid detection, characterized by their high flexibility, sensitivity and specificity.

**Methods:**

Combined recombinase polymerase amplification and CRISPR/Cas12a systems were combined and used as end-point diagnostic methods (termed REPORT) to detect *G. duodenalis* assemblage A and B. The diagnostic results can be observed by fluorescence readouts with the naked eye under blue light or colorimetric signals using a lateral flow strip (LFS).

**Results:**

The limit of detection (LOD) of the REPORT‑based *G. duodenalis* assemblage A detection was 2.04 CFU/ml and 10 trophozoites per gram (TPG), and the LOD of assemblage B was 1.1 CFU/ml and 10 cysts per gram (CPG). The REPORT‑based *G. duodenalis* assemblage A and assemblage B detection methods have strong specificity and no cross-reactivity with other assemblages of *G. duodenalis* or common enteric parasitic protozoa and have excellent performance in clinical sample detection.

**Conclusions:**

This study presents a novel strategy for the direct identification of *G. duodenalis* assemblages A and B, requiring neither highly trained personnel nor costly specialized equipment.

**Graphical Abstract:**

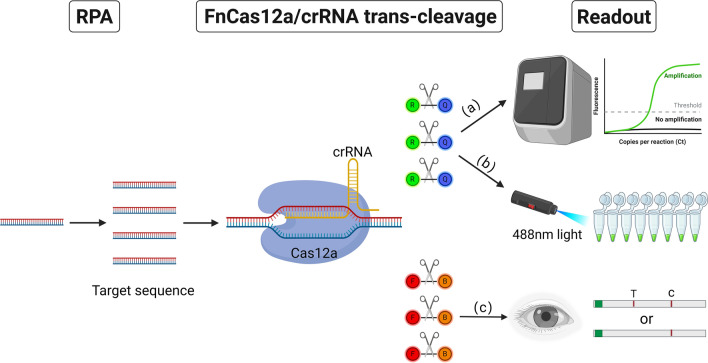

**Supplementary Information:**

The online version contains supplementary material available at 10.1186/s13071-024-06559-0.

## Background

*Giardia duodenalis* is one of the most common enteric protozoan parasites and infects humans and over 40 animal species [1]. Asymptomatic giardiasis frequently occurs in immunocompetent individuals; however, more severe symptoms like aqueous diarrhea, abdominal pain, weight loss, nutrient malabsorption and even death are often observed in immunocompromised patients [2, 3]. Infection with *G. duodenalis* happens through ingestion of contaminated food or water or via the fecal-oral route through host-to-host contacts [4]. The World Health Organization (WHO) reports that approximately 200 million people worldwide are infected with this disease [5]. Currently, at least eight assemblages (A–H) of *G. duodenalis* have been identified, with assemblages A and B recognized as zoonotic and documented in both humans and various animal species [6].

Currently, available techniques such as microscopy, immunology-based assays, polymerase chain reaction (PCR) and quantitative PCR (qPCR) have multiple drawbacks, including being time- and labor-intensive, lacking specificity and sensitivity [7–9] and requiring expensive equipment and well-trained personnel [10–12]. Novel methods such as loop-mediated isothermal amplification (LAMP) and recombinase polymerase amplification (RPA) operate under isothermal conditions, making them most suitable for on-site detection [13, 14]. Nevertheless, there is a pressing need for detection methods that are more sensitive, time-efficient, labor-saving and visual and less dependent on instrumentation for routine laboratory and field tests [15].

Clustered Regularly Interspaced Short Palindromic Repeats (CRISPR)/CRISPR-associated (CRISPR/Cas) systems provide bacteria and archaea with adaptive immunity against invading nucleic acids [16, 17]. Cas12 and Cas13, members of the Cas family, are capable of generating collateral cleavage of DNA and RNA, respectively [18–20]. When combined with RPA pre-amplification, Cas13 and Cas12a nucleases have been utilized to develop the Specific High-sensitivity Enzymatic Reporter UnLOCKing (SHERLOCK) system and the DNA Endonuclease-Targeted CRISPR Trans Reporter (DETECTR) system for highly sensitive and specific nucleic acid detection [18, 21]. In the CRISPR/Cas12a system, the CRISPR RNA (crRNA) specifically binds to target nucleic acids to form a ternary complex composed of Cas12a protein, guide RNA (gRNA) and the target nucleic acids. This complex exhibits robust collateral cleavage activity, indiscriminately cleaving surrounding nontarget single-stranded nucleic acids [20, 21]. The CRISPR/Cas12a system has been widely used to detect various pathogens such as *Cryptosporidium parvum* (*C. parvum*) [22], African swine fever virus (ASFV) [23], beta-coronavirus severe acute respiratory syndrome (SARS)-CoV-2 [24] and *Bacillus anthracis* [25]. In this study, a combined recombinase polymerase amplification and the CRISPR/Cas12a system (termed REPORT) detection technique was established by observing fluorescence readouts under blue light or using a lateral flow strip (LFS) biosensor to distinguish *G. duodenalis* assemblage A and assemblage B for rapid, specific, accurate and sensitive detection.

## Methods

### Sample information

The trophozoites of *G. duodenalis* assemblage A were obtained from Jilin University and cultured in our laboratory. The cysts of *G. duodenalis* assemblage B were purified from monkey feces collected at a zoo in Zhengzhou city, Henan Province. The DNA of *G. duodenalis* assemblages C–F, *C. parvum*, *Enterocytozoon bieneusi*, *Blastocystis hominis* (*B. hominis*) and *Entamoeba* species was stored in our laboratory. The DNA of assemblages C, D and F was obtained from dog and cat feces at a pet hospital in Zhengzhou city, Henan Province. The DNA of assemblage E, along with that of *C. parvum* and *E. bieneusi*, was obtained from dairy cattle feces on a farm in Zhengzhou, Henan Province. Sixty human fecal samples were collected from a hospital in Kafr El Sheikh Province, Egypt, and stored in our laboratory.

### Construction of a standard recombinant plasmid and plasmid DNA extraction

The complete sequences of the *tpi* gene from *G. duodenalis* assemblages A and B (GenBank accession nos. KM190791 and KP687783) were cloned into the pUC57 vector and subsequently transformed into *Escherichia coli* DH5α (Sangon Biotech, Shanghai, China). The transformed bacteria were cultured overnight in Luria-Bertani (LB) liquid medium at 37 °C with shaking at 180 rpm. Following this, the bacteria were serially diluted tenfold, and 100 μl of each dilution was inoculated onto LB solid medium, which was then incubated at 37 °C for 12 h. The bacterial concentration, expressed as colony-forming units per milliliter (CFU/ml), was determined by counting the colonies on the LB solid medium. Additionally, plasmid DNA was extracted using a rapid DNA extraction method, which involved cleaning and re-suspending the bacteria, incubating them at 95 °C for 10 min and then subjecting them to an ice bath for 2 min [26].

### *Giardia duodenalis* assemblage A trophozoite and assemblage B cyst counting and DNA extraction

*Giardia duodenalis* assemblage A trophozoites were cultured and counted according to a previously reported study [27]. Assemblage B cysts were purified from monkey fecal samples according to a previously reported study and counted using an Xb-k-25 Hemocytometer [28]. The purified trophozoites and cysts were mixed into the stool and extracted with E.Z.N.A.TM Stool DNA Kit purchased from Omega Bio-Tek Inc. (Norcross, GA, USA) following the instructions.

### Design and synthesis of crRNA

Four gene loci (*SSU* rRNA, β-giardin, glutamate dehydrogenase, triosephosphate isomerase) were commonly used in the identification of *G. duodenalis* [3]. Triosephosphate isomerase (*tpi*) gene was selected as the target for the design of crRNAs, because the assemblage-specific crRNAs were only screened at the *tpi* gene. Then, the 20–24 nucleotide (nt) sequence closely following a TTN protospacer adjacent motif (PAM) was selected as the target sequence, with a GC base content between 40 and 60%. A ‘synthetic mismatch’ was introduced into the crRNA-A1, which did not affect the crRNA's ability to recognize the target sequence of assemblage A, causing assemblage F to have two adjacent mismatched leads to off-target [29]. The T7 promoter (TAATACGACTCACTATAGGG) was utilized to generate the scaffold sequence of FnCas12a (AATTTCTACTGTTGTAGAT) and the target sequence (20–24 bp after the target PAM sequence) of crRNA-F, which exhibited inverse complementarity to crRNA-R, as previously described. The two single-stranded crRNA-F/Rs were synthesized by Sangon Biotech (Shanghai, China) and annealed to form double-stranded crRNA, which was transcribed using a HiScribe™ T7 High Yield RNA Synthesis Kit purchased from New England Biolabs (Ipswich, MA, USA), digested using Recombinant DNase I (RNase-free) purchased from TaKaRa Bio Inc. (Dalian, China) and purified by NucAway™ Spin Column purchased from Thermo Fisher Scientific, Inc. (Waltham, MA, USA) to obtain pure crRNA (Additional file: Table S1). The concentration of purified crRNA was measured using NanoDrop One (Thermo Fisher Scientific Inc., Waltham, MA, USA).

### Recombinase polymerase amplification assay

The concentration of the target sequence was increased by RPA using the TwistAmp® Basic kit purchased from TwistDx Ltd. (Hertfordshire, UK) according to the manufacturer’s instructions. The RPA primers were designed based on *tpi* gene using online NCBI Primer-BLAST (Additional file: Table S1) and synthesized by Sangon Biotech (Shanghai, China). The 50-μl reaction volume includes 29.5 μl rehydration buffer, 480 nM of each primer, 280 nM magnesium acetate (MgOAc), 5 μl template DNA and nuclease-free water. MgOAc was added to the tube lid, and other reagents were added directly to the tube, followed by instant centrifugation and vortex mixing. Finally, the reaction tube was placed in a constant temperature incubator at 37 °C for 30 min.

### FnCas12a/crRNA *trans*‑cleavage assay

The FnCas12a *trans*‑cleavage assay was performed as previously reported, with some optimization [21, 30]. The 20-μl reaction system included 50 nM FnCas12a (Tolo Biotech, Shanghai, China), 1 μM purified crRNA, 1.25 μM single-stranded DNA (ssDNA) probe [HEX-12N-BHQ1 reporter and FAM-12N-biotin reporter were synthesized by Sangon Biotech (Shanghai, China) and used for the fluorescence assay and the LFS assay], 2 μl 10 × FnCas12a nuclease reaction buffer, 2 μl target DNA (RPA products), 20 U RNase inhibitor (TaKaRa Bio Inc., Dalian, China) and DNase/RNase-free water (Beijing Solarbio Science & Technology Co., Ltd., China). Reaction conditions were maintained at 37 °C for 2 h. A qTOWER3G qPCR system (Analytik Jena, Germany) was used to record the fluorescence every 5 min and generate real-time fluorescence curves.

### Construction of the LFS assay

To achieve on-site diagnosis of *G. duodenalis* assemblages A and B, the LFS was used. The FAM-12N-biotin ssDNA reporter could specifically bind to an anti-FITC antibody conjugated with Au nanoparticles. When the *trans*‑cleavage function of FnCas12a was not activated by the target DNA, the complex (FAM-12N-biotin ssDNA reporter with anti-FITC antibody) was captured by the biotin ligand fixed on the control line. However, FnCas12a activated by the target DNA exerts its *trans*‑cleavage function to cut the ssDNA reporter, and the complex cannot be captured by the biotin ligand fixed on the control line but by the IgG antibody at the test line. Determination of test results was conducted as follows: if the control line on the test strip turned red while the detection line exhibited no color change, the test result was determined as negative. Conversely, if both the control and detection lines turned red, the test sample was classified as positive. Following the completion of the FnCas12a *trans*‑cleavage assay, 4 μl of the reaction mixture was combined with 196 μl of diluent; subsequently, 80 μl of this mixture was absorbed and added to the LFS. The LFS was then incubated at 25 °C for 7 to 10 min to allow for result observation.

### PCR amplification of the *G. duodenalis bg* gene

To evaluate the performance of the REPORT-based detection method, a nested PCR method based on the *bg* loci of *G. duodenalis*, according to a previous study, was used to detect and compare the same samples [31]. The primers used for the nested PCR are listed in Additional file: Table S1 and synthesized by Sangon Biotech (Shanghai, China). The 25 μl nested PCR reaction system included 1 × KOD-Plus buffer, 0.5 units KOD-Plus DNA polymerase (ToYoBo Co., Ltd., Osaka, Japan), 200 μM dNTPs, 500 nM of each primer, 1 mM MgSO4, 2 μl DNA template and ddH_2_O. The reaction procedure of the first round included pre-denaturation at 95 °C for 5 min and then 35 cycles of 94 °C for 35 s, 60 °C for 35 s and 72 °C for 1 min, with a final extension at 72 °C for 10 min. Annealing temperature of the second reaction was change from 60 to 55 °C, while the other conditions remained constant. The nested PCR products were added to a 1% agarose gel for electrophoresis, and the results were observed using a UV gel imager. The assemblage type of positive samples was confirmed by bidirectional sequencing (SinoGenoMax Biotechnology Co., Ltd. Beijing, China), aligning obtained sequences using Clustal X 2.1 (http://www.clusteral.org/), with reference sequences downloaded from GenBank.

### Statistical analysis

Statistical analyses were performed using GraphPad Prism 8 and presented as mean ± SD. *P* < 0.001 was considered to indicate a statistically significant difference.

## Results

### Design and preparation of crRNA

Download and align the *tpi* gene sequences of *G. duodenalis* assemblages A–H and other *Giardia* species to find 20–24 nt target sequences that are assemblage-conserved and specific relative to other assemblages and *Giardia* species. The complementary crRNA sequence was determined according to the target sequence, and then the corresponding two single-stranded crRNA-F/Rs were synthesized by annealing, transcription, DNase I digestion and NucAway™ Spin Column purification to obtain pure crRNA (Additional file: Table S1). The concentrations of all crRNAs measured by NanoDrop One were showed in Additional file: Figure S1.

### Screening of optimal RPA primer and crRNA

**Fig. 1:**
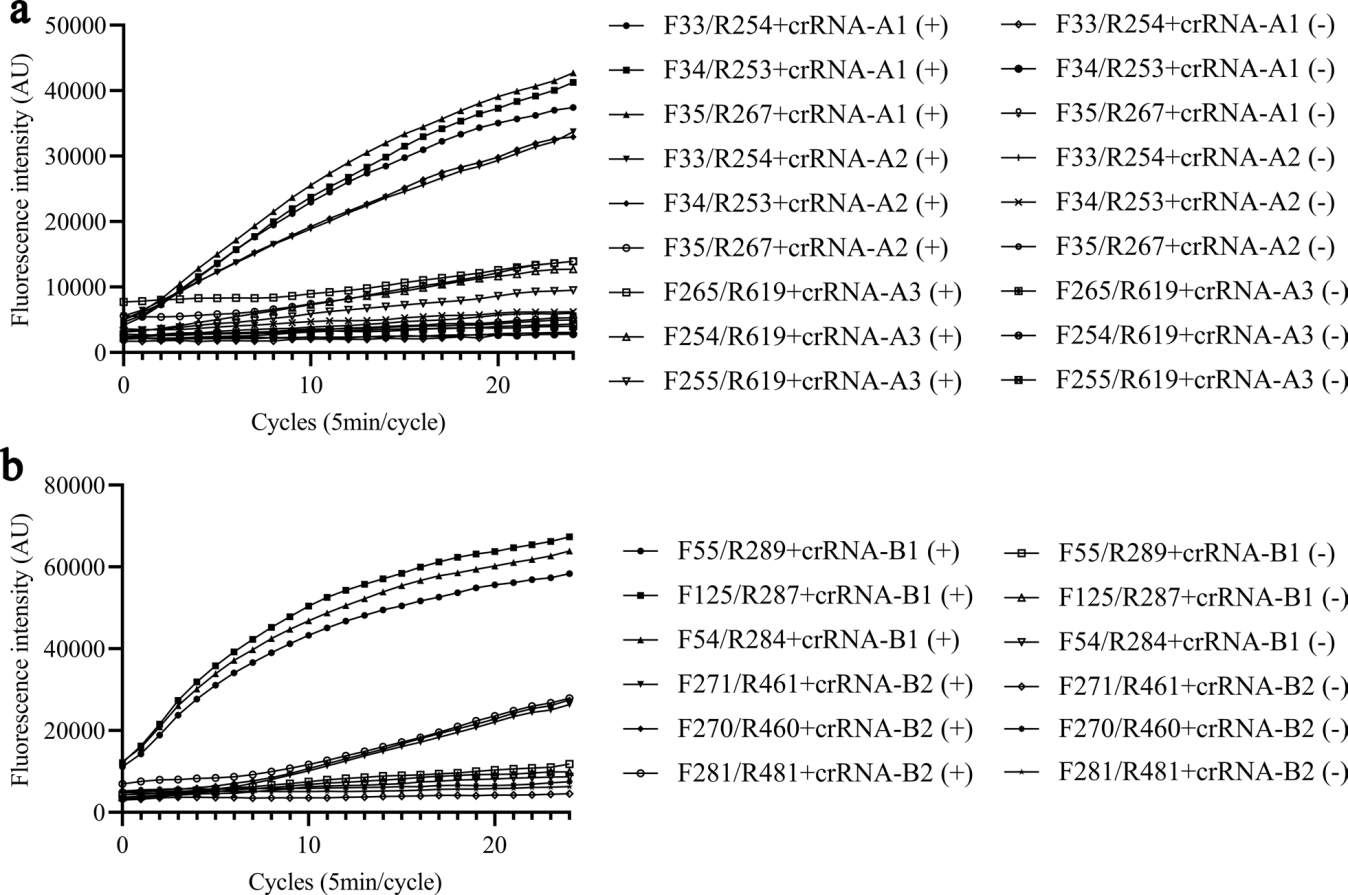
Screening for optimal primer pairs for RPA. Primer pairs listed in Table S1 were also tested. Primer pair F35/R267 and crRNA-A1 for *Giardia duodenalis* assemblage A and primer pair F125/R287 and crRNA-B1 for *G. duodenalis* assemblage B were found to be the best

Several RPA primers were designed based on the *tpi* gene of *G. duodenalis* assemblage A (GenBank accession No. KM190791), and three crRNAs were selected based on the position of the RPA primers. The RPA and FnCas12a/crRNA *trans*-cleavage assays were performed using the above RPA primers and crRNA-A, respectively. The results showed that the F35/R267 primers with crRNA-A1 had the highest fluorescence intensity; therefore, they were chosen as the best primers and crRNA (Fig. 1a). Similarly, the RPA primers and crRNAs for *G. duodenalis* assemblage B were designed based on the *tpi* gene of *G. duodenalis* (GenBank accession No. KP687783) and screened using RPA and FnCas12a/crRNA *trans*-cleavage assays. The results indicated that the best primer and crRNA for *G. duodenalis* assemblage B were F125/R287 and crRNA-B1. (Fig. 1b).

### Buffer optimization of the FnCas12a/crRNA *trans*-cleavage assay

**Fig. 2:**
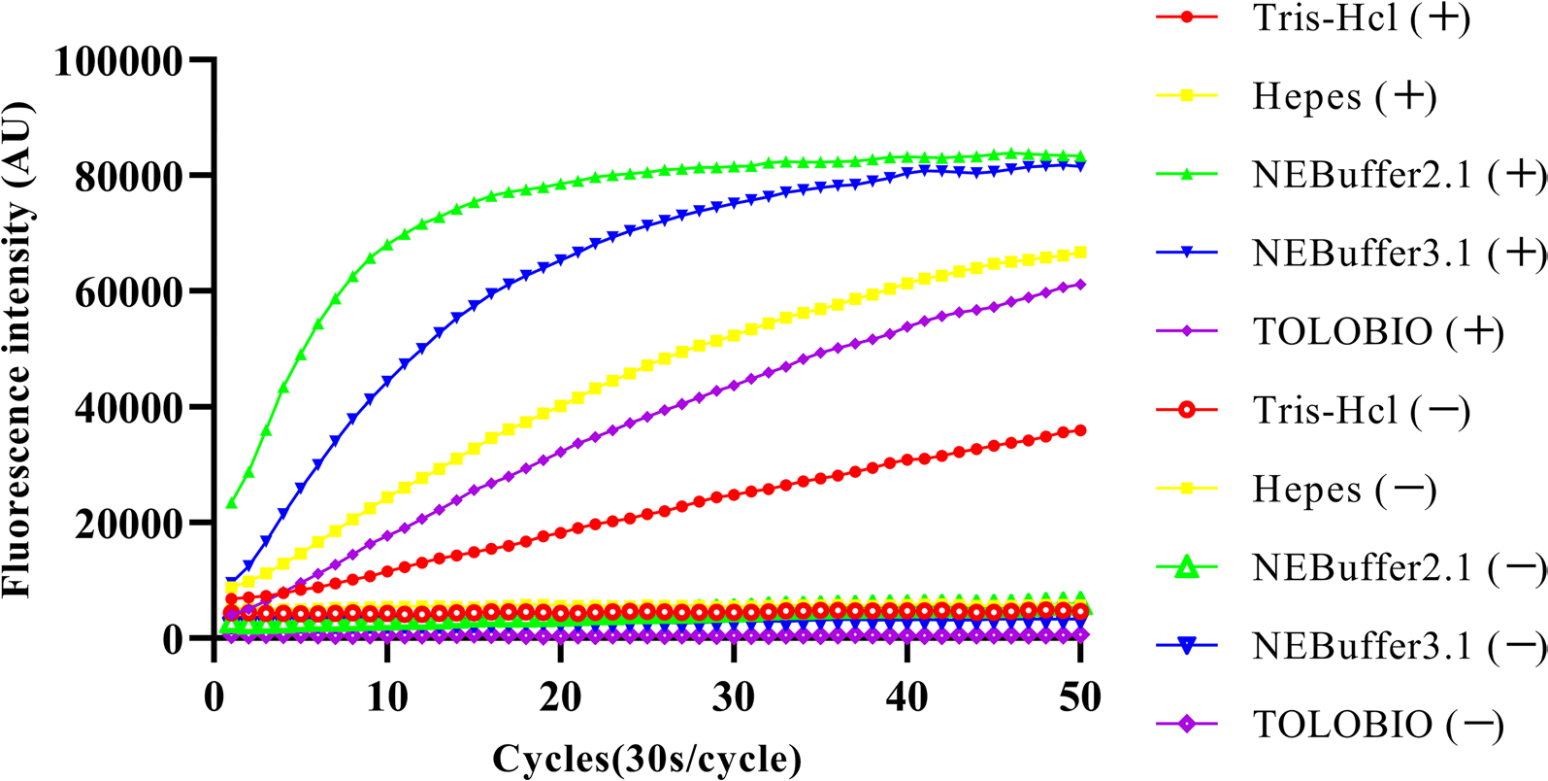
Optimization buffer for the FnCas12a/crRNA trans cleavage assay. Tris–HCl ( +), Hepes (+), NEBuffer 2.1 (+), NEBuffer 3.1 (+) and Tolobio (+) were positive DNA as sample. Tris–HCl (−), Hepes (−), NEBuffer 2.1 (−), NEBuffer 3.1 (−) and Tolobio (−) were ddH_2_O as samples

The buffer plays a significant role in the FnCas12a/crRNA *trans*‑cleavage assay, and a good buffer can speed up the reaction. To have a better detection performance, Tolobio buffer (purchased along with FnCas12a), Tris–HCl (20 mmol/l Tris–HCl pH 8.0, 100 mmol/l KCl, 5 mmol/l MgCl_2_, 50 μg/ml heparin, 1 mmol/l DTT, 5% glycerinum), Hepes (20 mmol/l Hepes pH8.0, 100 mmol/l KCl, 5 mmol/l MgCl_2_, 50 μg/ml heparin, 1 mmol/l DTT, 5% glycerinum), NEBuffer 2.1 and NEBuffer 3.1 were respectively selected as reaction buffer to incubate at 37 °C for 25 min. The result showed that when the reaction buffer was NEBuffer 2.1, the *trans*‑cleavage reaction reached the maximum fluorescence intensity in the shortest time (Fig. 2). Therefore, NEBuffer 2.1 was selected as the FnCas12a/crRNA *trans*‑cleavage assay reaction buffer.

### Optimization of the concentration of LFS reporter

**Fig. 3:**
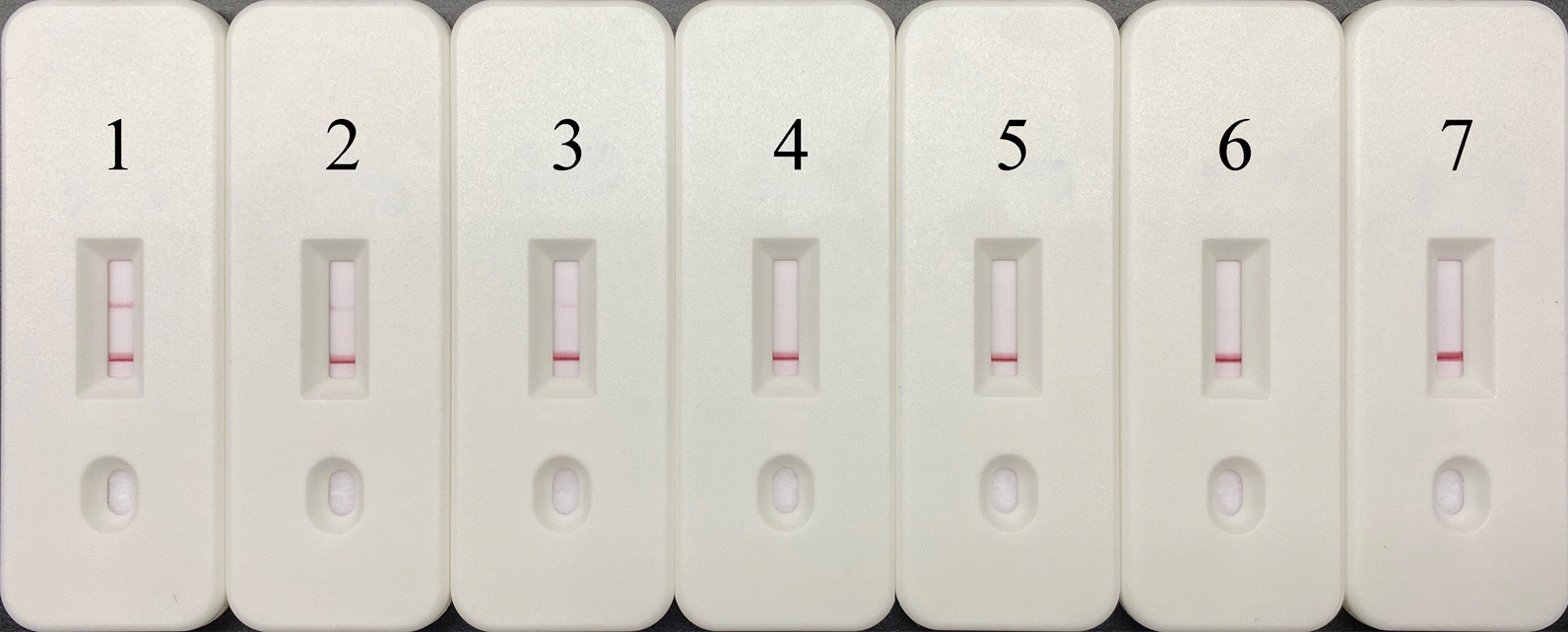
Optimization of reporter concentration for REPORT-based LFS detection. Various concentrations of the FAM-12N-biotin ssDNA reporter were tested to avoid false-positive results. 1–7: 5 nM, 10 nM, 15 nM, 20 nM, 30 nM, 40 nM and 50 nM FAM-12N-biotin ssDNA reporter

In the LFS assay, when the concentration of the FAM-12N-biotin ssDNA reporter is low, the Au nanoparticles will not completely combine to the anti-FITC antibody, and then free Au nanoparticles flowing forward and being captured by the anti-FAM secondary antibody at the test line will lead to a false-positive result. To avoid this, five different concentrations of the FAM-12N-biotin ssDNA reporter between 5 and 50 nM were tested on LFS without interference from any target DNA samples. The test results showed that false-positive results would appear when the concentration of FAM-12N-biotin ssDNA reporter was ≤ 15 nM and disappear when the concentration of FAM-12N-biotin ssDNA reporter was ≥ 20 nM (Fig. 3). Therefore, the concentration of the FAM-12N-biotin ssDNA reporter was determined to be 20 nM in REPORT-based LFS detection.

### Feasibility verification of REPORT‑based detection

**Fig. 4:**
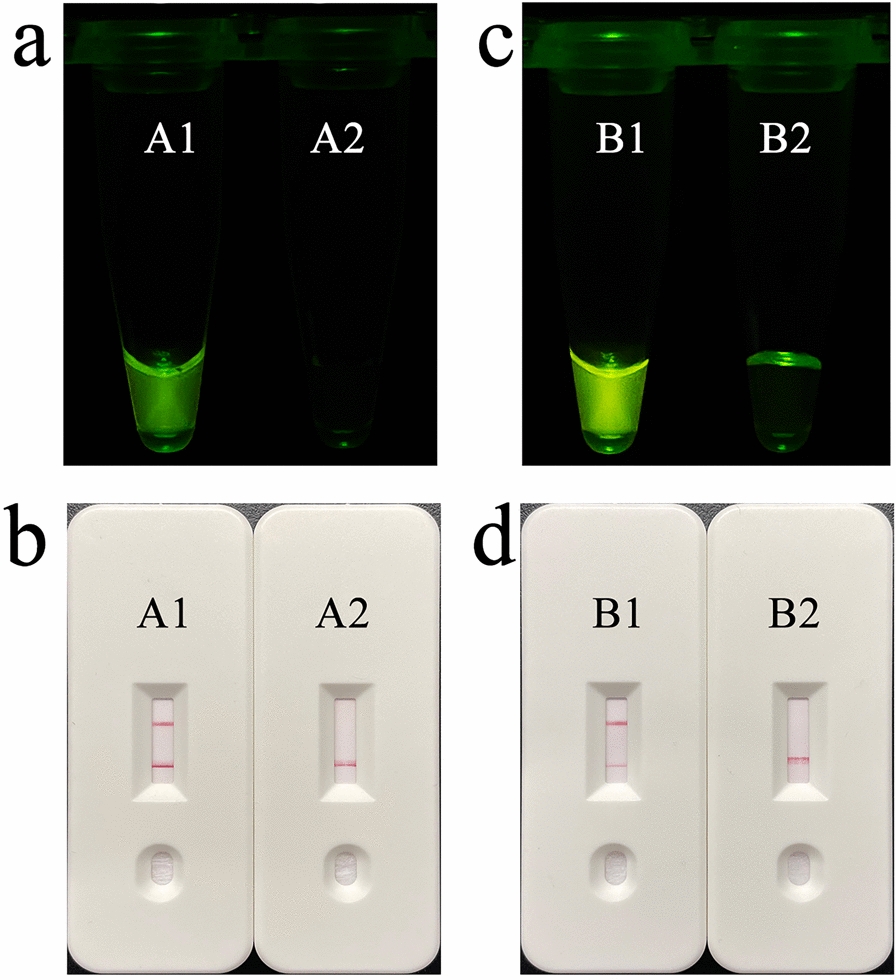
Feasibility verification of the REPORT-based detection. **a** Obvious fluorescence signal can be observed under UV light by the naked eye for assemblage A. A1: positive results, A2: positive results. **b** An obvious test line can be observed in the LFS of assemblage A. **c** An obvious fluorescence signal can be observed under UV light by the naked eye in assemblage B. B1: positive results, B2: positive results. **d** An obvious test line can be observed in the LFS of assemblage B

Based on the REPORT system, this study aimed to establish a detection technique to distinguish *G. duodenalis* assemblage A and assemblage B, and the results could be read out by fluorescence and test strips. The designed crRNA with FnCas12a binds to the target dsDNA to form a triplex, which activates FnCas12a. Next, the activated FnCas12a will perform *trans*-cleavage to cleave the HEX-12N-BHQ1 reporter to emit 520-nm fluorescence under 488-nm light or cleave the FAM-12N-biotin reporter to display a test line on the LFS. Using the positive DNA samples of *G. duodenalis* assemblages A or B as templates, RPA and enzyme digestion were performed using F35/R267 primers and crRNA-A1, F125/R287 primers and crRNA-B1, respectively. The CRISPR/Cas12a-based fluorescence detection assay was placed under a blue light instrument. The products of positive samples emitted clear, visibly detectable fluorescence, which was significantly different from that of the negative control (Fig. 4a and c). In the LFS assay, a red line visible to the naked eye appeared in the test line on the LFS of the positive sample. In contrast, no color change was observed in the test line of the negative sample on the LFS (Fig. 4b and d).

### Specificity of the REPORT‑based detection

**Fig. 5:**
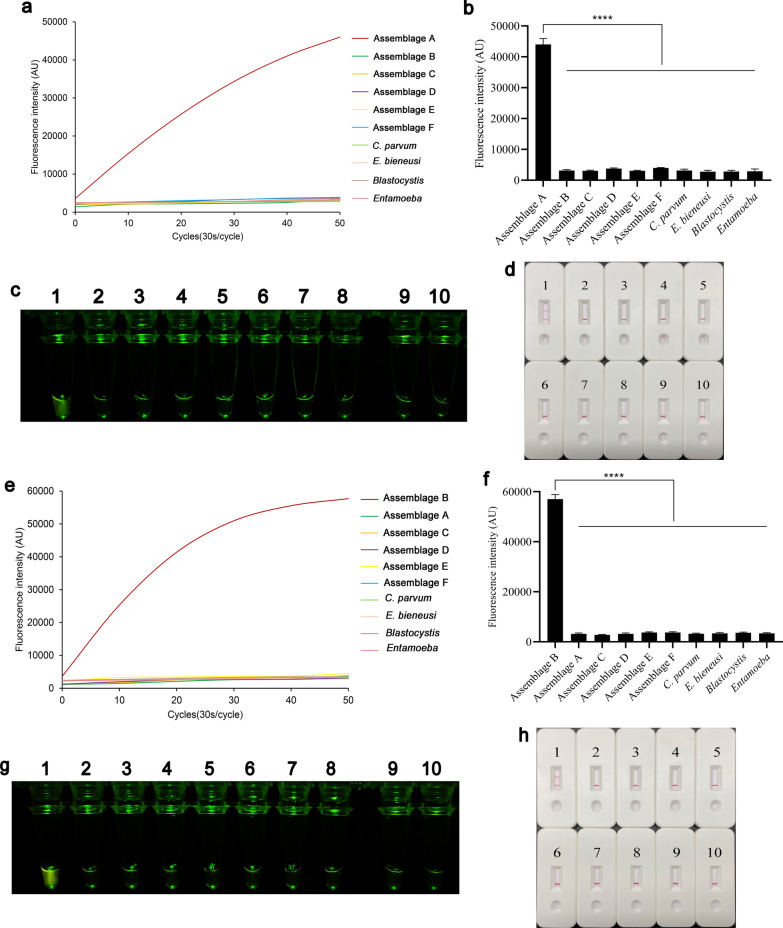
Specificity of REPORT-based detection. The specificity of REPORT-based *Giardia duodenalis* assemblage A detection method was assessed using real-time fluorescence signals (**a**), quantitative analysis (**b**) (****P < 0.0001; bars represent the means ± SEMs) and visible green fluorescence (**c**). **d** Specificity of the REPORT-based assemblage A LFS detection for nine pathogen-positive DNA. 1–10: *G. duodenalis* assemblages A–F, *Cryptosporidium parvum*, *Enterocytozoon bieneusi*, *Blastocystis hominis* and *Entamoeba*, respectively. The specificity of REPORT-based *G. duodenalis* assemblage B detection method was assessed using real-time fluorescence signals (**e**), quantitative analysis (**f**) (****P < 0.0001, bars represent the means ± SEMs) and visible green fluorescence (**g**). **h** Specificity of the REPORT-based assemblage B LFS detection for nine pathogen-positive DNA. 1–10: *G. duodenalis* assemblage B, assemblage A, assemblages C–F, *C. parvum*, *E. bieneusi*, *B. hominis* and *Entamoeba*, respectively

Genomic DNA extracted from six different *G. duodenalis* assemblages (assemblages A–F) and other common intestinal protozoa (*C. parvum*, *E. bieneusi*, *B. hominis*, *Entamoeba*) were used to verify the specificity of the REPORT‑based assemblage A detection method and assemblage B detection method. In the REPORT‑based assemblage A detection method, only *G. duodenalis* assemblage A showed high fluorescence intensity differences from other parasites (*P* < 0.0001) (Fig. 5a and b). The *G. duodenalis* assemblage A emitted a visible green fluorescence and test line that could be distinguished by the naked eye (Fig. 5c and d). Similarly, the result of the REPORT‑based assemblage B detection method showed that assemblage B had high fluorescence intensity (*P* < 0.0001) (Fig. 5e and f), a visible green fluorescence and test line different from other parasites (Fig. 5g and 5h).

### Sensitivity of the REPORT‑based detection (plasmid DNA)

**Fig. 6:**
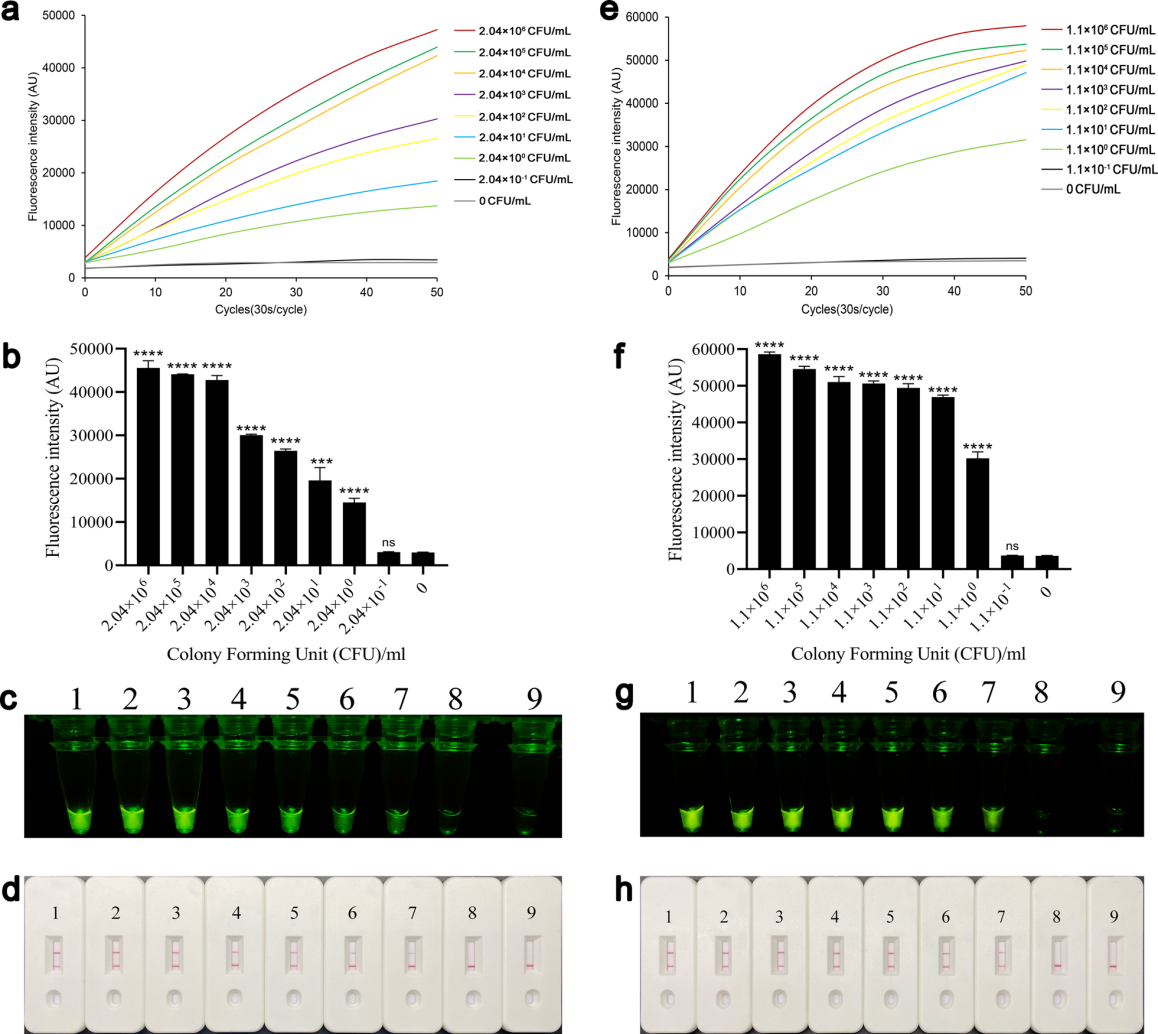
Sensitivity of the REPORT-based detection of plasmid DNA. The sensitivity of REPORT-based *Giardia duodenalis* assemblage A detection method was assessed using real-time fluorescence signals (**a**), quantitative analysis (**b**) (****P < 0.0001, the bars represent the means ± SEMs) and visible green fluorescence (**c**). **d** Sensitivity of the REPORT-based assemblage A LFS detection for various concentrations of plasmid DNA. 1–9: 2.04 × 10^6^–2.04 × 10^–1^ CFU/ml, 0 CFU/ml. The sensitivity of REPORT-based *G. duodenalis* assemblage B detection method was assessed using real-time fluorescence signals (**e**), quantitative analysis (**f**) (****P < 0.0001; the bars represent the means ± SEMs) and visible green fluorescence (**g**). **h** Sensitivity of the REPORT-based assemblage B LFS detection for various concentrations of plasmid DNA, 1–9: 1.1 × 10^6^–1.1 × 10^–1^ CFU/ml, 0 CFU/ml

The complete sequences of *G. duodenalis* assemblages A and B *tpi* were cloned into the pUC57 plasmid, which was introduced into *E. coli* DH5α. The bacteria expanded the culture and plate count to assess the limit of detection (LOD) of REPORT‑based detection. The plasmid DNA was extracted, serially diluted to different concentrations and then applied to REPORT‑based detection. The bacteria concentration (assemblages A) was 2.04 × 10^8^ CFU/ml, and it was successively diluted from 2.04 × 10^8^ CFU/ml to 2.04 × 10^–1^ CFU/ml. In the sensitivity test of the REPORT‑based *G. duodenalis* assemblage A detection method, the results showed that when the concentration of the sample was ≥ 2.04 × 10^0^ CFU/ml, there were high fluorescence intensity differences from 2.04 × 10^–1^ CFU/ml and negative control (*P* < 0.0001) (Fig. 6a and b). Also, the visible green fluorescence and test line could be distinguished by the naked eye in 2.04 × 10^6^–2.04 × 10^0^ CFU/ml (Fig. 6c and d). The bacteria concentration (assemblage B) was 1.1 × 10^8^ CFU/ml, and it was successively diluted from 1.1 × 10^8^ CFU/ml to 1.1 × 10^–1^ CFU/ml. In the sensitivity test of the REPORT‑based assemblage B detection method, the results showed that when the concentration of sample was 1.1 × 10^0^ CFU/ml or higher, there were high fluorescence intensity differences from 1.1 × 10^–1^ CFU/ml and negative control (*P* < 0.0001) and a visible green fluorescence and a test line different from 1.1 × 10^–1^ CFU/ml and negative control (Fig. 6g and h).

### Sensitivity of the REPORT‑based detection (trophozoite or cyst)

**Fig. 7:**
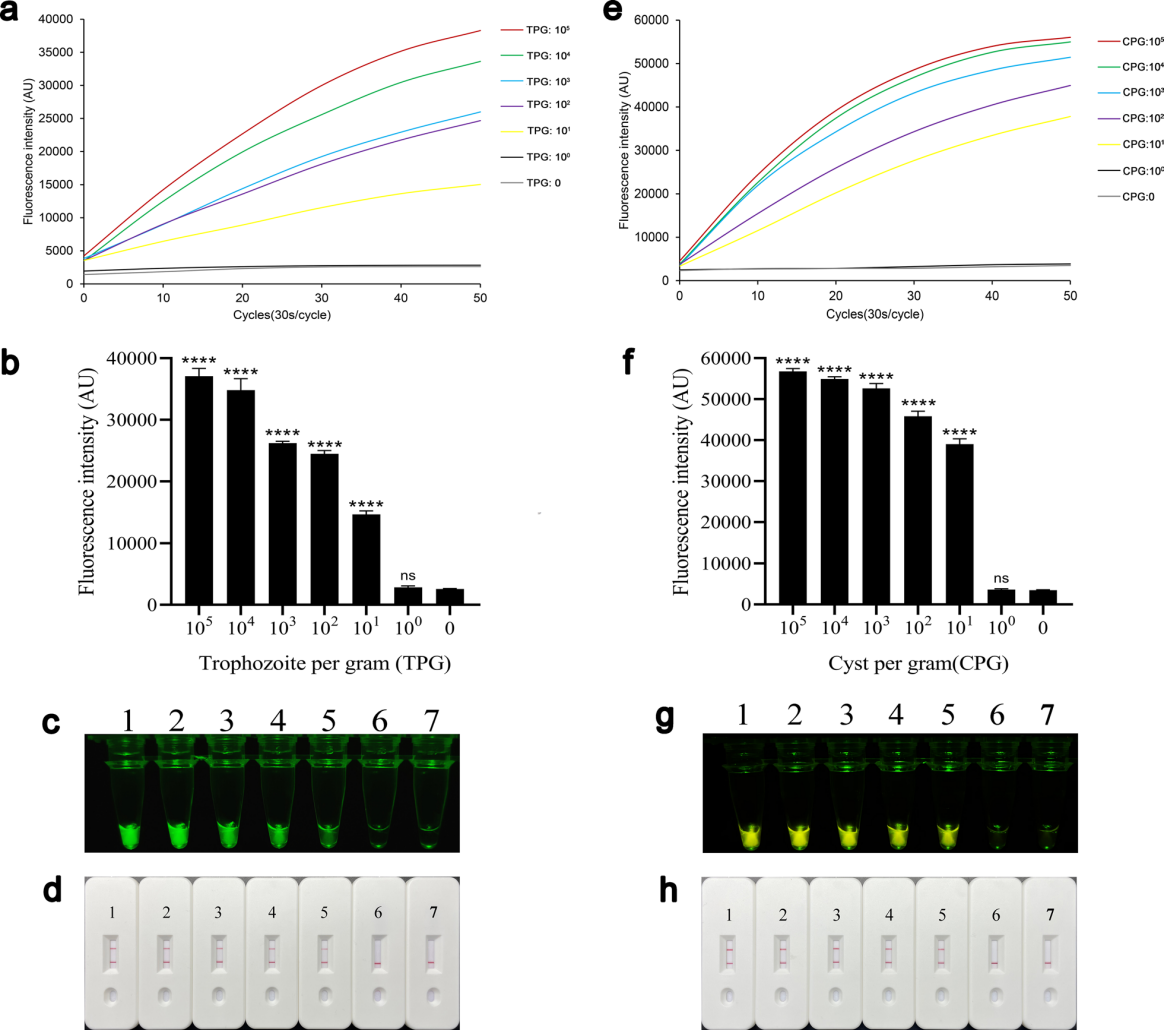
Sensitivity of REPORT-based detection of trophozoites and cysts. The sensitivity test of REPORT-based *Giardia duodenalis* assemblage A detection method was assessed using real-time fluorescence signals (**a**) and quantitative analysis (**b**) (****P < 0.0001; the bars represent the means ± SEMs) and visible green fluorescence (**c**). **d** Sensitivity test of the REPORT-based assemblage A LFS detection for various concentrations of genomic DNA. 1–7: 10^5^–10^0^ TPG, 0 TPG. The sensitivity test of REPORT-based *G. duodenalis* assemblage B detection method was assessed using real-time fluorescence signals (**e**) and quantitative analysis (**f**) (****P < 0.0001, the bars represent the means ± SEMs) and visible green fluorescence (**g**). **h** Sensitivity test of the REPORT-based assemblage B LFS detection for various concentrations of genomic DNA, 1–7: 10^5^–10^0^ CPG, 0 CPG

Genomic DNA was extracted from clinical-simulated positive samples of *G. duodenalis* for sensitivity testing and REPORT‑based detection. The pure cultured trophozoites of *G. duodenalis* assemblage A were counted and mixed into the feces to 10^5^ trophozoites per gram of feces (TPG:10^5^), and then the genomic DNA was extracted. In the sensitivity test of the REPORT-based assemblage A detection method, the samples of 10^5^–10^1^ TPG have high fluorescence intensity compared to the samples of 10^0^ TPG and negative control (*P* < 0.0001) (Fig. 7a and b). Also, the samples of 10^5^–10^1^ TPG have emitted a visible green fluorescence and test line that could be distinguished by the naked eye (Fig. 7c and d). The purified cysts of assemblage B were counted and mixed into the feces to 10^5^ cysts per gram of feces (CPG:10^5^), and then the genomic DNA was extracted. In the sensitivity test of the REPORT-based assemblage B detection mothed, the samples of 10^5^–10^1^ CPG have high fluorescence intensity compared to the samples of 10^0^ CPG and negative control (*P* < 0.0001) (Fig. 7e and f). Also, the samples of 10^5^–10^1^ CPG emitted a visible green fluorescence and test line that could be distinguished by the naked eye in the 10^0^ CPG sample and negative control (Fig. 7g and h).

### Performance of the REPORT‑based *G. duodenalis* assemblage A and assemblage B detection methods on clinical samples

Sixty human fecal samples from Egypt, known to be positive and negative, were used to assess the clinical performance of REPORT-based *G. duodenalis* assemblage A and assemblage B detection methods. The samples were amplified using nested PCR based on the *bg* gene. The results of agarose gel electrophoresis showed that 26 samples were amplified with the 510-bp target bands (Additional file: Figure S2). The sequencing result showed that 23 samples confirmed positive for *G. duodenalis* and 3 samples (sample 7, sample 21 and sample 42) failed to be sequenced because the target product fragment concentration was too low. The infection rate of *G. duodenalis* assemblage A was 15.0% (9/60), of *G. duodenalis* assemblage B was 20.0% (12/60) and of assemblages A and B mixed infection was 3.3% (2/60) (Additional file: Table S2).

**Fig. 8:**
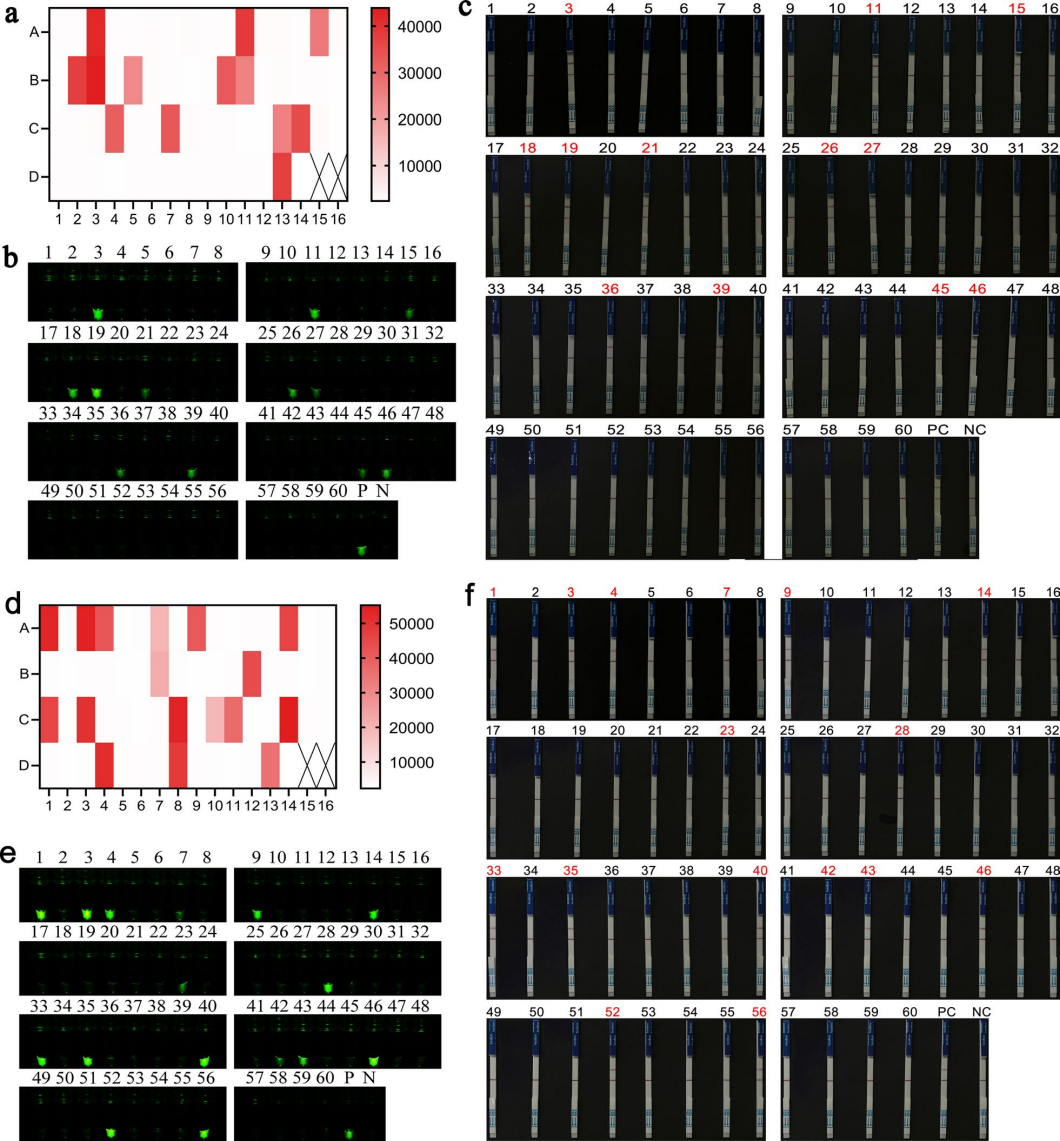
Validation of the REPORT-based detection of *Giardia duodenalis* assemblage A and assemblage B in human clinical samples. In the REPORT‑based assemblage A detection method, 11 PCR-positive samples of assemblage A and 1 sequencing-failed sample (sample 21) had fluorescence intensity (**a**), visible green fluorescence (**b**) and a test line (**c**). In the REPORT‑based assemblage B detection method, 14 PCR-positive samples of assemblage B and 2 sequencing-failed samples (sample 7 and 42) had fluorescence intensity (**a**), visible green fluorescence (**b**) and a test line (**c**). 1–60: 60 human samples; PC: positive control; NC: negative control

Sixty samples were respectively detected by REPORT-based *G. duodenalis* assemblage A and assemblage B detection methods. In the REPORT‑based assemblage A detection method, 11 PCR-positive samples of assemblage A and 1 sequencing-failed sample (sample 21) had fluorescence intensity and visible green fluorescence (Fig. 8a and d). In the REPORT‑based assemblage B detection method, 14 PCR-positive samples of assemblage B and 2 sequencing-failed samples (sample 7 and 42) had fluorescence intensity and visible green fluorescence (Fig. 8d and e). The results of the REPORT-based LFS detection consistent with the results of REPORT‑based fluorescence detection showed that all PCR-positive samples and three sequencing-failed samples (sample 7, 21 and 42) had a test line (Fig. 8c and f).

## Discussion

In this study, RPA and the Cas12a/crRNA *trans*-cleavage system were combined to establish a REPORT-based detection technique to distinguish *G. duodenalis* assemblage A and assemblage B. The result can be obtained from the fluorescence signal or the LFS. The results showed that the REPORT-based detection technique to distinguish *G. duodenalis* assemblage A and assemblage B has high sensitivity and specificity. CRISPR/Cas12a has been widely used to detect *Cryptosporidium parvum* [22], ASFV [23] and SARS-CoV-2 [24]. In this study, the CRISPR/Cas12a system was applied to specifically detect *G. duodenalis* assemblage A and assemblage B for the first time to our knowledge. The REPORT-based fluorescence or LFS assay showed some advantages in point-of-care use without the need for expensive equipment, time consumption and technical expertise.

The CRISPR/Cas12a biosensing system generally includes three important components: signal amplification, signal conversion and signal reporting [32]. For signal amplification, the RPA was selected for its high amplification efficiency and consistent reaction temperatures. Recombinase polymerase amplification and Cas12a *trans* cleavage were also conducted at 37 °C, which made REPORT more convenient. A water or metal bath, a constant temperature incubator or even body temperature can be used to carry out REPORT-based detection.

Signal conversion was realized by the Cas12a/crRNA *trans*‑cleavage system, which can convert the presence of target DNA into fluorescence or colorimetric signals [32]. When the target DNA was present, the high endonuclease activity of FnCas12a was activated, which cut the ssDNA reporter [30]. Combined with RPA, REPORT-based detection is highly sensitive owing to the efficient cleavage activity of FnCas12a.

In this study, signal reporting included fluorescence readout and LFS, corresponding to the HEX-12N-BHQ1 and FAM-12N-biotin probes, respectively. In the Cas12a/crRNA *trans*‑cleavage assay, when the HEX-12N-BHQ1 probe was used, the fluorescence signals were observed by the naked eye under blue light with a Tanon-5200 Multi Fluorescence Imager. In resource-poor areas, inexpensive blue light meters are a good option [33]. When the FAM-12N-BHQ1 probe was used, the result could be observed by the LFS without any equipment, which makes the REPORT-based LFS detection truly useful for *G. duodenalis* assemblage A and B detection under field conditions.

There are limitations to the REPORT-based detection technique to distinguish *G. duodenalis* assemblage A and assemblage B established in this study. First, the detection technique requires two sample addition operations: RPA amplification and FnCas12a/crRNA *trans*-cleavage assay, which increases the risk of cross-contamination. Integrating the two reactions in a single reaction tube to create a “one-pot” assay would be an improvement. Second, some reagents used in this method need to be stored at − 20 ℃, and there are certain restrictions when they are used in some areas. All reagents being premixed and freeze-dried would expand the application range of the detection technique.

In conclusion, the recombinase polymerase amplification and CRISPR/Cas12a systems were combined to establish a detection technique to distinguish *G. duodenalis* assemblage A and assemblage B (termed REPORT). The results can be observed by fluorescence readouts with the naked eye under blue light or colorimetric signals with LFS in on-site diagnosis. The REPORT-based detection technique established in this study could distinguish *G. duodenalis* assemblage A and assemblage B in clinical fecal samples without professional technicians and expensive instruments within approximately 70 min. At the same time, the REPORT-based detection methods demonstrated high sensitivity in both pure and complex samples, with strong specificity also being confirmed. The REPORT-based detection technique to distinguish *G. duodenalis* assemblage A and assemblage B is superior to the nested PCR sequencing method based on the *bg* gene, which is commonly used to diagnose *G. duodenalis*. Further optimization of the REPORT assay as a one-pot reaction should be pursued in future research to better facilitate the rapid and straightforward detection of *G. duodenalis* assemblage A or assemblage B from clinical samples in the field settings.

## Supplementary Information

Additional file 1: Table S1. Nucleotide sequences were used in this study. Table S2. Sequencing results for positive products. Figure S1. Absorbance curves of purified crRNA. The crRNAs were transcribed from the crDNA annealed using two reverse complementary single-strand oligonucleotides. The transcribed crRNAs were treated with DNase I and purified using a NucAway™ Spin Column. Figure S2. Results of *Giardia duodenalis* nested PCR amplification based on the *bg* locus. 1–60: Human samples; P: positive control; N: negative control.

## Data Availability

The data supporting the findings of this study are available from the corresponding author, Longxian Zhang: zhanglx8999@henau.edu.cn.

## Abbreviations

Cas: CRISPR-associated
CRISPR: Clustered regularly interspaced short palindromic repeats
crRNA: CRISPR RNA
gRNA: Guide RNA
dsDNA: Double-stranded DNA
*bg*: β-Giardin
LFS: Lateral flow strip
LOD: Limit of detection
PAM: Protospacer-adjacent motif
PCR: Polymerase chain reaction
REPORT: Recombinase polymerase amplification and CRISPR/Cas12a system
TPG: Trophozoites per gram
CPG: Cysts per gram
RPA: Recombinase polymerase amplification
ssDNA: Single-stranded DNA
LAMP: Loop-mediated isothermal amplification
SHERLOCK: The Specific High-sensitivity Enzymatic Reporter UnLOCKing
DETECTR: The DNA Endonuclease-Targeted CRISPR Trans Reporter
ASFV: African swine fever virus
(SARS)-CoV-2: Beta-coronavirus severe acute respiratory syndrome
*tpi*: Triosephosphate isomerase
nt: Nucleotide
