# Supplementary material for: End-point diagnostics of Giardia duodenalis assemblages A and B by combining RPA with CRISPR/Cas12a from human fecal samples
Source: Parasit Vectors. 2024 Nov 12;17:463. doi: 10.1186/s13071-024-06559-0 (PMC11558988; doi:10.1186/s13071-024-06559-0)
Supplement: Supplementary file 1 — Additional file 1: Table S1. Nucleotide sequences were used in this study. Table S2. Sequencing results for positive products. Figure S1. Absorbance curves of purified crRNA. The crRNAs were transcribed from the crDNA annealed using two reverse complementary single-strand oligonucleotides. The transcribed crRNAs were treated with DNase I and purified using a NucAway™ Spin Column. Figure S2. Results of Giardia duodenalis nested PCR amplification based on the bg locus. 1–60: Human samples; P: positive control; N: negative control. [file 13071_2024_6559_MOESM1_ESM.docx]

Supplemental Materials

**End-point diagnostics of *Giardia duodenalis* assemblages A and B by combining CRISPR/Cas12a with RPA from human fecal samples**

Yilin Wang^a,b^, Fuchang Yu^a,b,c^, Yin Fu^a,b^, Qian Zhang^d^, Jinfeng Zhao^a,b^, Ziyang Qin^a,b^, Ke Shi^e^, Yayun Wu^a,b^, Junqiang Li^a,b^, Xiaoying Li^a,b^, Longxian Zhang^a,b,^*

^a^ *College of Veterinary Medicine, Henan Agricultural University, Zhengzhou, Henan, P. R. China*

^b^ *National International Joint Research Center for Animal Immunology, Zhengzhou, Henan, P. R. China*

^c^ *College of Animal Science and Technology, Tarim University, Alar, Xinjiang 843300, PR China*

^d^ *YEBIO Bioengineering Co.,Ltd of QINGDAO, Qingdao, Shandong, 266108, PR China*

^e^ *School of Medicine, Xinxiang University, Jinsui Road 191, Xinxiang, 453003, China*

* Corresponding author

College of Veterinary Medicine, Longzihu Campus of Henan Agricultural University, No. 15 Longzihu University Area, Zhengzhou New District, Zhengzhou, 450046, P. R. China.

Tel.: 86-371-56990163; Fax: 86-371-56990163;

E-mail: zhanglx8999@henau.edu.cn

**TABLE S1** Nucleotide sequences used in this study

| Name | | Sequence (5’-3’) |
| --- | --- | --- |
| Target (assemblage A) | | TACACCTGTCAACAGCCATTGCGGCAA |
| Target (assemblage B) | | GAAGTGTCTGAAAATAGCAGCACAGAA |
| RPA primers (assemblage A) | |  |
| 1 | F33 | CTTCAAGTGTAACGGCTCTCTTGACTTTATC |
|  | R254 | CTACTATCACATGCTTCAAACCCATGTCCTG |
| 2 | F34 | TTCAAGTGTAACGGCTCTCTTGACTTTATC |
|  | R253 | TACTATCACATGCTTCAAACCCATGTCCTGA |
| 3 | F35* | TCAAGTGTAACGGCTCTCTTGACTTTATCA |
|  | R267* | CTTTCAGAGTGCCCTACTATCACATGCTTCA |
| 4 | F265 | TTTGAAGCATGTGATAGTAGGGCACTCTGAAA |
|  | R619 | GATCCCCCGTAAATGATACGGATATGCTGTG |
| 5 | F254 | CAGGACATGGGTTTGAAGCATGTGATAGTAG |
|  | R619 | GATCCCCCGTAAATGATACGGATATGCTGTG |
| 6 | F255 | AGGACATGGGTTTGAAGCATGTGATAGTAG |
|  | R619 | GATCCCCCGTAAATGATACGGATATGCTGTG |
| RPA primers (assemblage B) | |  |
| 1 | F55 | GACTTCATTAAGAGCCACGTAGCGTCCATCG |
|  | R289 | GTCTCGCCCATGATTCTACGTCTTTCAGAG |
| 2 | F125* | CTCCCTCCTTTGTGCACCTTTCTACAGCTA |
|  | R287* | CTCGCCCATGATTCTACGTCTTTCAGAGTG |
| 3 | F54 | CGACTTCATTAAGAGCCACGTAGCGTCCATC |
|  | R284 | GCCCATGATTCTACGTCTTTCAGAGTGTCC |
| 4 | F271 | CCATGTAATAATAGGACACTCTGAAAGACG |
|  | R461 | CTCCCATAACTTCTTTGATTCTCCAATCTC |
| 5 | F270 | GCCATGTAATAATAGGACACTCTGAAAGAC |
|  | R460 | CTCCCATAACTTCTTTGATTCTCCAATCTCC |
| 6 | F281 | ATAGGACACTCTGAAAGACGTAGAATCATGG |
|  | R481 | CTCATAGGCAATTACAACGTTCTCCCATAAC |
| PCR primers | |  |
| BG1 | | AAGCCCGACGACCTCACCCGCAGTGC |
| BG2 | | GAGGCCGCCCTGGATCTTCGAGACGAC |
| BG3 | | GAACGAACGAGATCGAGGTCCG |
| BG4 | | CTCGACGAGCTTCGTGTT |
| crRNA | |  |
| crRNA-A-F1* | | gaaatTAATACGACTCACTATAgggAATTTCTACTGTTGTAGATccgcaatggctgttgacaggtata |
| crRNA-A-R1* | | tatacctgtcaacagccattgcggATCTACAACAGTAGAAATTcccTATAGTGAGTCGTATTAatttc |
| crRNA-A-F2 | | gaaatTAATACGACTCACTATAgggAATTTCTACTGTTGTAGATaggatagcagcgcagaatgtgtac |
| crRNA-A-R2 | | gtacacattctgcgctgctatcctATCTACAACAGTAGAAATTcccTATAGTGAGTCGTATTAatttc |
| crRNA-A-F3 | | gaaatTAATACGACTCACTATAgggAATTTCTACTGTTGTAGATgactctccgagctccttgccaa |
| crRNA-A-R3 | | ttggcaaggagctcggagagtcATCTACAACAGTAGAAATTcccTATAGTGAGTCGTATTAatttc |
| crRNA-B-F1* | | gaaatTAATACGACTCACTATAgggAATTTCTACTGTTGTAGATtgtgctgctattttcagacacttc |
| crRNA-B-R1* | | gaagtgtctgaaaatagcagcacaATCTACAACAGTAGAAATTcccTATAGTGAGTCGTATTAatttc |
| crRNA-B-F2 | | gaaatTAATACGACTCACTATAgggAATTTCTACTGTTGTAGATtccagagcacgcttcgccttctta |
| crRNA-B-R2 | | taagaaggcgaagcgtgctctggaATCTACAACAGTAGAAATTcccTATAGTGAGTCGTATTAatttc |

Underline indicates the PAM region.

* Corresponds to the optimum RPA primers and crRNA used in this study

**Table S2.** Sequencing results for positive products.

| Assemblage type | Sample No. |
| --- | --- |
| Assemblage A | 11, 15, 18, 19, 26, 27, 36, 39, 45 |
| Assemblage B | 1, 4, 9, 14, 23, 28, 33, 35, 40, 43, 52, 56 |
| A and B mixed | 3, 46 |
| Sequencing failed | 7, 21, 42 |


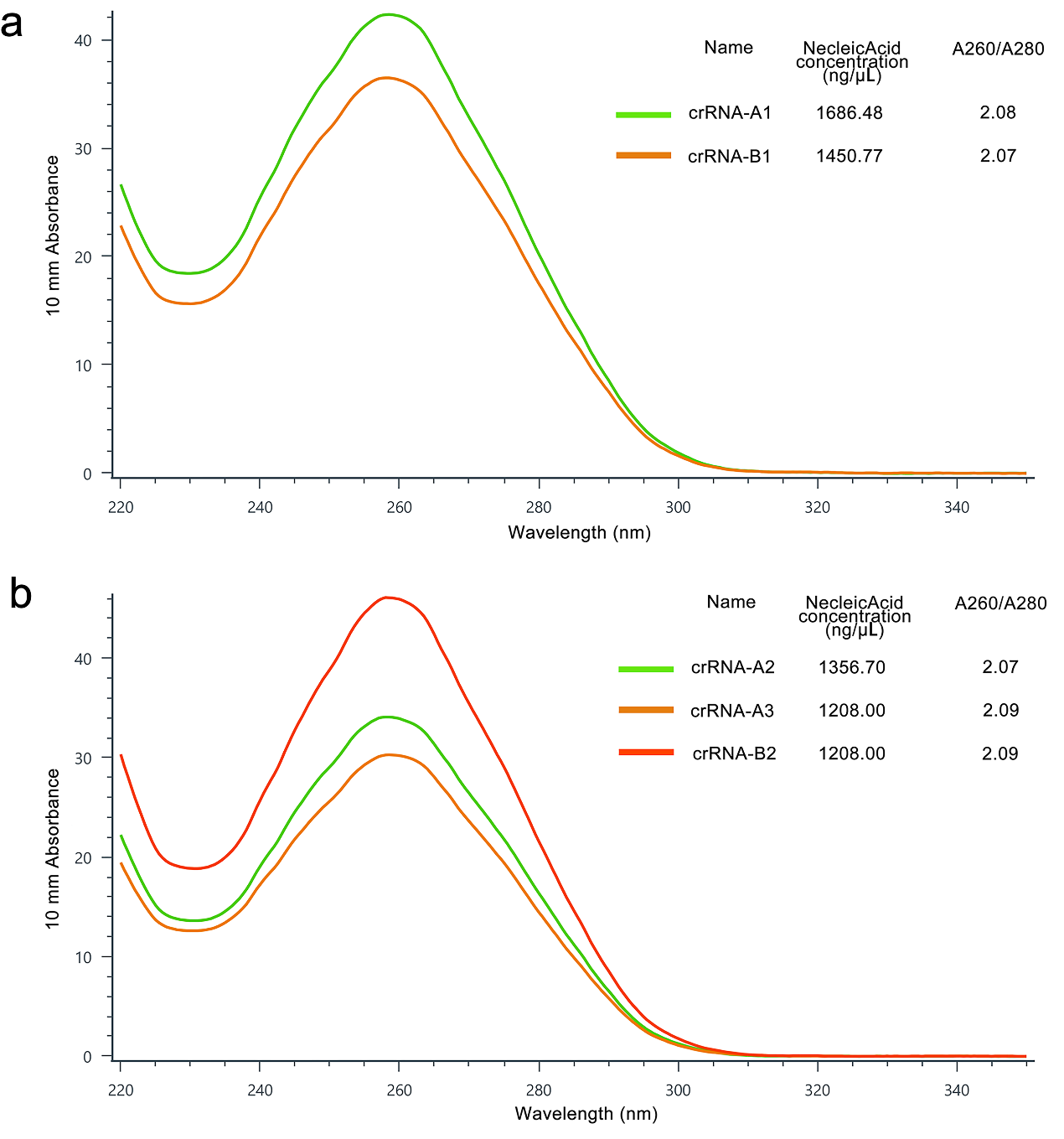


**Figure. S1. Absorbance curves of purified crRNA.** The crRNAs were transcribed from the crDNA annealed using two reverse complementary single-stranded oligonucleotides. The transcribed crRNAs were treated with DNase I and purified using a NucAway™ Spin Column.


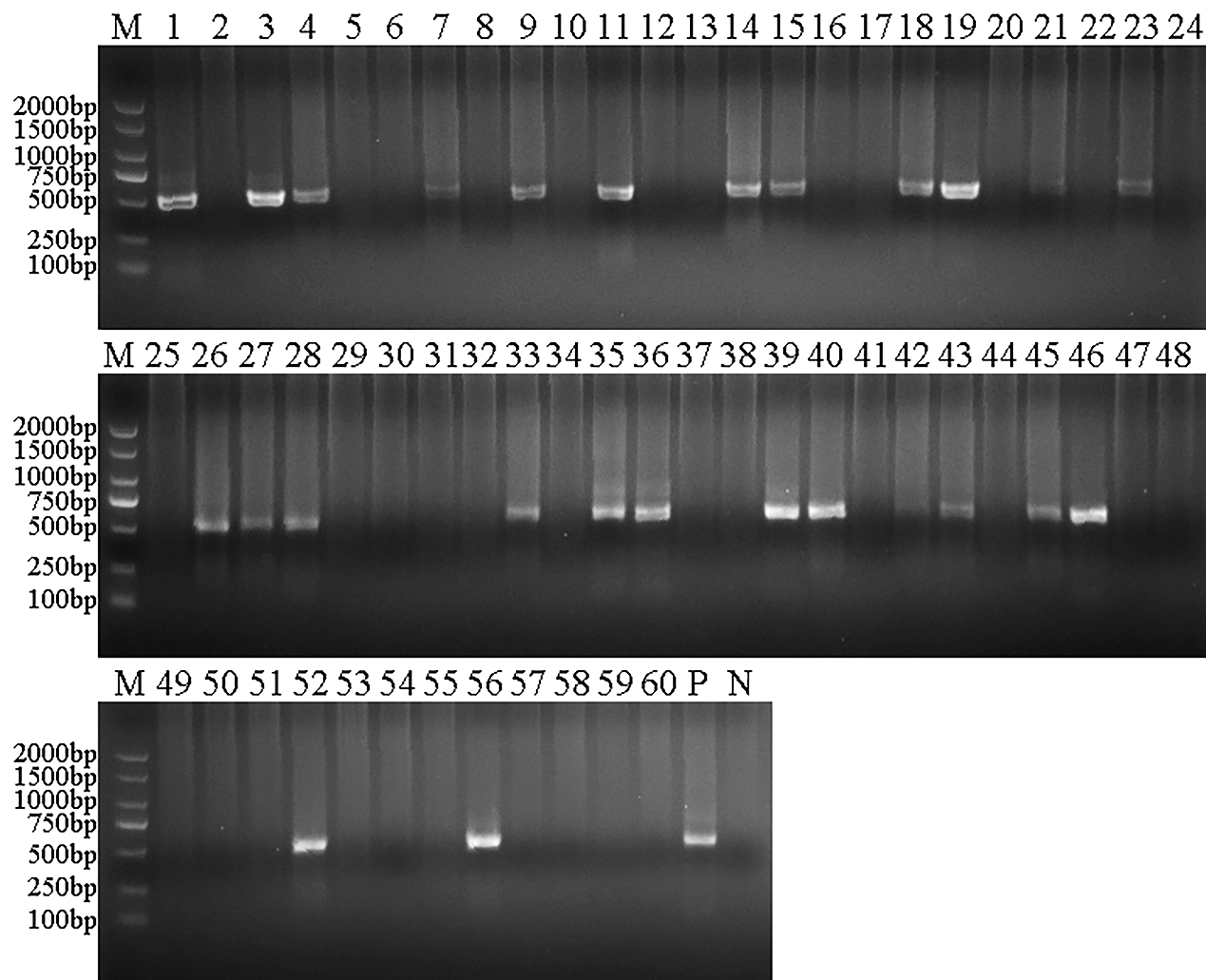


**Fig S2. Results of *G. duodenalis* nested PCR amplification based on the *bg* locus.** 1-60: Human samples; P: Positive control; N: Negative control.
